# Supplementary material for: A Likelihood Approach for Real-Time Calibration of Stochastic Compartmental Epidemic Models
Source: PLoS Comput Biol. 2017 Jan 17;13(1):e1005257. doi: 10.1371/journal.pcbi.1005257 (PMC5240920; doi:10.1371/journal.pcbi.1005257)
Supplement: S1 File — (TAR.GZ) [file pcbi.1005257.s014.tar.gz › HSPH_Online-SI-Revision/EnKF07/n1-severe/n1-severe_EnKF_table8.pdf]

1	R0	{1.51081, 13.7078}	{2.69734, 8.60154}	{{1.83546, 2.21824}, {3., 14.3076}}	{78.5363, 39.2397}	inf	54.	73.664	{15.1003, 84.2755}	128.228	0.399376	newly	28.	59.5	{9., 52.}	175.714	0.326527	Reff	0.70857	0.443151	{0.13261, 0.64904}	53.9052	ar	656.	1672.97	{1495., 1977.}	155.026
2	R0	{1.73182, 11.3873}	{2.25144, 4.05693}	{{0.300662, 0.159899}, {3., 8.22113}}	{74.9455, 72.532}	inf	39.	64.7011	{6.2416, 87.7923}	108.281	0.31604	newly	19.	74.71	{8., 132.}	316.263	0.501493	Reff	0.680605	0.355223	{0.0440979, 0.481875}	62.1871	ar	653.	1655.5	{1503., 1901.}	153.522
3	R0	{1.50449, 10.3474}	{2.8482, 6.94964}	{{2.42726, 2.2917}, {3., 8.98604}}	{90.4742, 40.1212}	inf	38.	76.8241	{7.23341, 196.152}	151.664	0.408858	newly	22.	57.65	{5., 157.}	200.591	0.414605	Reff	0.664985	0.407735	{0.0509911, 0.611508}	64.2791	ar	651.	1786.11	{1589., 2295.}	174.364
4	R0	{1.63493, 9.1955}	{2.66504, 6.33072}	{{2.11346, 3.40641}, {3., 14.8519}}	{66.4798, 60.1017}	inf	17.	34.3043	{5.21792, 70.2259}	136.655	0.327205	newly	9.	37.77	{6., 104.}	339.	0.456786	Reff	0.614734	0.509829	{0.0874036, 1.11358}	60.6225	ar	663.	1658.78	{1526., 1858.}	150.193
5	R0	{1.53822, 8.05553}	{2.52043, 4.02764}	{{1.77722, 0.897738}, {3., 11.1253}}	{63.8538, 63.7347}	inf	26.	76.269	{5.32621, 162.024}	223.356	0.417455	newly	17.	127.54	{7., 362.}	673.412	0.616827	Reff	0.642976	0.572648	{0.107141, 1.55594}	67.2793	ar	621.	1748.56	{1504., 2345.}	181.572
6	R0	{1.69374, 17.0793}	{2.60863, 6.6928}	{{1.62616, 3.59862}, {3., 15.4569}}	{56.114, 60.8134}	inf	76.	25.2173	{6.9891, 30.9909}	79.8113	0.696205	newly	35.	27.36	{4., 29.}	85.0286	0.472188	Reff	0.753714	0.40548	{0.0668554, 0.710347}	55.4537	ar	662.	1525.11	{1419., 1637.}	130.379
7	R0	{1.42179, 11.8031}	{2.56661, 7.91025}	{{1.58741, 3.24755}, {3., 19.3555}}	{80.5197, 52.053}	inf	20.	74.7719	{8.34236, 197.113}	297.977	0.409766	newly	9.	78.96	{8., 171.}	788.222	0.583666	Reff	0.720848	0.610808	{0.0980053, 1.58582}	57.1285	ar	517.	1754.16	{1483., 2558.}	239.296
8	R0	{1.46683, 6.95875}	{2.66439, 6.19909}	{{2.01492, 0.992066}, {3., 14.2788}}	{85.1886, 56.752}	inf	0.1	62.042	{7.50476, 84.4952}	61942.	2.47858	newly	0.1	89.74	{6., 130.}	89644.	2.4254	Reff	0.50899	0.475896	{0.124986, 1.0239}	60.9405	ar	653.	1712.45	{1537., 2182.}	162.243
9	R0	{1.34525, 11.505}	{2.59958, 9.091}	{{1.66049, 2.47146}, {3., 15.8314}}	{93.2417, 46.5661}	inf	36.	60.1299	{10.0767, 89.4724}	105.035	0.312911	newly	22.	60.27	{8., 109.}	210.409	0.359833	Reff	0.768138	0.595523	{0.14376, 1.37452}	51.0254	ar	532.	1666.07	{1478., 1840.}	213.171
10	R0	{1.77958, 14.372}	{2.55911, 5.86724}	{{1.61872, 0.997849}, {3., 13.8238}}	{49.4634, 60.8423}	inf	31.	58.1026	{5.95958, 96.9349}	133.727	0.362451	newly	16.	66.98	{6., 75.}	349.5	0.498836	Reff	0.619294	0.522692	{0.0981317, 0.692795}	43.8277	ar	671.	1607.47	{1461., 1718.}	139.563
11	R0	{1.60334, 19.9548}	{2.65787, 4.01317}	{{1.75906, 2.2007}, {3., 8.70682}}	{65.771, 79.8887}	inf	70.	24.8127	{2.06606, 33.51}	89.6749	0.856835	newly	28.	27.65	{2., 39.}	89.9643	0.493288	Reff	0.80167	0.515759	{0.119685, 0.918373}	45.3049	ar	617.	1525.42	{1428., 1635.}	147.232
12	R0	{1.68623, 7.58585}	{2.77079, 8.54174}	{{2.43184, 3.60794}, {3., 16.9873}}	{65.6775, 48.8643}	inf	7.	28.2951	{3.26566, 24.3718}	323.94	0.50319	newly	7.	21.24	{2., 27.}	230.857	0.428894	Reff	0.612101	0.495844	{0.15488, 0.845656}	54.5762	ar	640.	1635.63	{1518., 1784.}	155.567
13	R0	{1.82689, 18.0022}	{2.6691, 3.17833}	{{1.62881, 1.17953}, {3., 5.22365}}	{51.2861, 82.7452}	inf	56.	37.8518	{3.53708, 40.732}	102.446	0.715685	newly	15.	58.44	{4., 78.}	328.	0.468509	Reff	0.668642	0.48517	{0.0756009, 0.633131}	45.1233	ar	673.	1582.99	{1458., 1934.}	135.214
14	R0	{1.75911, 9.16883}	{2.71924, 10.3631}	{{2.45351, 4.02949}, {3., 18.4181}}	{57.3779, 53.1664}	inf	25.	87.0416	{9.30328, 114.818}	267.67	0.383788	newly	15.	71.91	{6., 97.}	403.933	0.423327	Reff	0.656148	0.553076	{0.259115, 0.622759}	52.8901	ar	661.	1791.52	{1566., 2470.}	171.032
15	R0	{1.46168, 11.3859}	{2.35419, 7.8873}	{{0.774161, 0.509191}, {3., 18.6335}}	{88.3096, 59.0502}	inf	9.	101.625	{8.44464, 311.481}	1035.16	0.803557	newly	3.	97.23	{9., 221.}	3141.67	1.11418	Reff	0.654833	0.328761	{0.102725, 0.455774}	55.1084	ar	556.	1752.71	{1517., 2342.}	215.236
16	R0	{1.53577, 11.7581}	{2.67196, 6.43978}	{{2.44625, 1.47142}, {3., 13.4703}}	{80.6241, 55.0601}	inf	31.	50.3292	{7.17556, 61.522}	102.453	0.29521	newly	13.	60.32	{5., 78.}	384.462	0.471334	Reff	0.664988	0.57736	{0.0901276, 0.957275}	59.1729	ar	625.	1693.84	{1529., 1977.}	171.014
17	R0	{1.70698, 9.72924}	{2.79559, 8.86284}	{{2.4681, 3.40629}, {3., 13.8627}}	{63.7742, 49.0703}	inf	8.	85.1195	{8.96067, 159.052}	970.908	0.760735	newly	4.	112.28	{7., 384.}	2714.5	0.964809	Reff	0.571838	0.762059	{0.360995, 1.85724}	61.361	ar	675.	1807.42	{1535., 2497.}	167.766
18	R0	{1.5973, 14.931}	{2.2079, 10.9905}	{{0.064271, 5.01806}, {3., 18.8987}}	{83.9852, 39.5012}	inf	59.	90.3906	{14.0262, 77.6226}	115.186	0.326603	newly	28.	71.55	{8., 76.}	211.179	0.369498	Reff	0.967964	0.400103	{0.0106511, 0.666991}	68.5659	ar	632.	1744.71	{1565., 1945.}	176.062
19	R0	{1.87468, 17.004}	{2.47842, 4.23041}	{{0.411208, 1.05969}, {3., 5.39497}}	{61.8708, 75.1211}	inf	48.	13.5927	{1.48305, 22.2195}	80.8499	0.808503	newly	12.	21.74	{1., 25.}	149.333	0.423943	Reff	0.684258	0.356372	{0.0403146, 0.520833}	69.9267	ar	692.	1503.04	{1430., 1523.}	117.202
20	R0	{1.84715, 12.531}	{2.83883, 5.74674}	{{2.32847, 1.61677}, {3., 12.6301}}	{53.6872, 54.5234}	inf	9.	64.8807	{8.08944, 74.908}	629.667	0.653207	newly	3.	127.64	{9., 642.}	4156.	1.15707	Reff	0.676057	0.671349	{0.154126, 1.12344}	49.3957	ar	661.	1676.43	{1465., 2374.}	153.62
21	R0	{1.75938, 13.5959}	{2.77863, 3.90926}	{{2.47812, 2.72429}, {3., 6.55808}}	{57.9321, 71.2468}	inf	38.	24.6586	{3.40925, 40.5634}	94.7518	0.624391	newly	12.	41.68	{3., 64.}	289.167	0.411017	Reff	0.777646	0.552566	{0.171048, 1.03403}	53.8867	ar	620.	1683.14	{1534., 2057.}	171.474
22	R0	{1.63456, 10.7469}	{2.28048, 4.97319}	{{0.601696, 1.37095}, {3., 9.2654}}	{77.2341, 55.5518}	inf	11.	91.8135	{9.63873, 316.969}	746.096	0.646522	newly	7.	105.8	{7., 435.}	1420.86	0.796223	Reff	0.568827	0.382911	{0.100111, 0.575629}	37.6881	ar	654.	1650.34	{1433., 2569.}	152.346
23	R0	{1.62702, 19.1392}	{2.84434, 5.89616}	{{2.28875, 0.673906}, {3., 15.3607}}	{74.8188, 69.2632}	inf	42.	34.3564	{4.96533, 60.8252}	87.4677	0.494072	newly	16.	81.93	{5., 364.}	460.063	0.42035	Reff	0.715889	0.576223	{0.172111, 1.34925}	64.1936	ar	600.	1663.97	{1507., 2023.}	177.328
24	R0	{1.70466, 12.1202}	{2.52946, 3.88905}	{{0.735522, 1.30735}, {3., 5.37082}}	{68.8611, 67.9127}	inf	15.	22.0737	{2.43941, 31.1865}	115.936	0.357971	newly	6.	38.09	{2., 34.}	556.5	0.463337	Reff	0.685273	0.471454	{0.0938742, 0.500029}	64.0202	ar	613.	1553.18	{1444., 1697.}	153.374
25	R0	{1.61735, 11.3169}	{2.58612, 7.57247}	{{1.29906, 5.32304}, {3., 10.086}}	{69.9183, 33.087}	inf	17.	30.4606	{2.07462, 25.68}	123.992	0.351003	newly	11.	26.22	{2., 40.}	185.273	0.453306	Reff	0.533726	0.46039	{0.0637613, 0.729417}	53.1847	ar	681.	1494.57	{1381., 1656.}	119.467
26	R0	{1.56977, 12.3035}	{2.49599, 4.97664}	{{1.5961, 0.384827}, {3., 14.3332}}	{60.4585, 65.9753}	inf	23.	64.6325	{9.78614, 138.72}	204.897	0.332898	newly	15.	72.07	{6., 138.}	404.067	0.522635	Reff	0.758199	0.520307	{0.1361, 1.03963}	56.5276	ar	594.	1733.13	{1529., 2301.}	191.773
27	R0	{1.46317, 5.8298}	{2.79484, 5.10506}	{{1.80057, 2.14473}, {3., 7.83409}}	{92.6596, 34.0902}	inf	0.1	56.6757	{6.68634, 73.5994}	56575.7	2.40951	newly	0.1	48.77	{6., 84.}	48670.	2.37451	Reff	0.605752	0.375854	{0.146849, 0.58936}	52.7397	ar	586.	1677.46	{1516., 2029.}	186.256
28	R0	{1.70002, 14.9806}	{2.75773, 9.03234}	{{2.38319, 3.08019}, {3., 12.7776}}	{64.4404, 39.7064}	inf	50.	53.4366	{8.35607, 36.0605}	101.03	0.435562	newly	31.	33.72	{4., 44.}	93.0323	0.409427	Reff	0.683408	0.548183	{0.201659, 0.926908}	52.7471	ar	652.	1709.1	{1557., 2032.}	162.132
29	R0	{1.52729, 16.1014}	{2.85374, 6.85167}	{{2.36409, 2.68564}, {3., 11.0711}}	{86.85, 57.4468}	inf	25.	48.1854	{3.43393, 37.7204}	152.32	0.399348	newly	10.	43.78	{3., 66.}	370.2	0.511501	Reff	0.720881	0.609006	{0.0821203, 1.5719}	63.1558	ar	592.	1622.77	{1428., 2273.}	174.117
30	R0	{1.72795, 13.5635}	{2.2439, 7.00839}	{{0.544273, 2.62888}, {3., 15.7208}}	{71.731, 54.1962}	inf	17.	80.5378	{6.75818, 338.491}	403.016	0.421781	newly	2.	60.09	{4., 225.}	2904.5	1.06312	Reff	0.60651	0.398111	{0.0750091, 0.526747}	55.8968	ar	668.	1755.54	{1514., 2694.}	162.805
31	R0	{1.51591, 18.1544}	{2.7661, 4.91794}	{{2.18184, 2.19042}, {3., 7.77737}}	{83.1202, 73.2895}	inf	38.	91.098	{16.3845, 325.267}	173.986	0.305794	newly	24.	127.66	{14., 410.}	451.	0.425085	Reff	0.858005	0.518403	{0.100467, 1.29206}	67.5799	ar	541.	1719.55	{1496., 2383.}	217.847
32	R0	{1.4282, 10.3534}	{2.47541, 4.61124}	{{0.263793, 2.63265}, {3., 6.88511}}	{104.346, 57.2978}	inf	37.	21.3259	{2.6098, 41.6311}	77.6969	0.598645	newly	27.	26.53	{3., 62.}	103.074	0.575195	Reff	0.854064	0.437506	{0.0475262, 1.0738}	69.1045	ar	521.	1578.12	{1474., 1868.}	202.902
33	R0	{1.56693, 11.3972}	{2.01301, 6.89392}	{{0.0400254, 0.197204}, {3., 15.0675}}	{84.2798, 51.5694}	inf	21.	59.5585	{5.58221, 80.1526}	216.464	0.37419	newly	13.	48.26	{5., 87.}	306.	0.438198	Reff	0.518654	0.396313	{0.00630875, 0.70646}	65.9515	ar	695.	1699.38	{1536., 2042.}	144.515
34	R0	{1.50675, 19.8823}	{2.75351, 3.69095}	{{1.88891, 1.73515}, {3., 4.5459}}	{82.7452, 81.436}	inf	31.	35.0896	{3.35316, 18.749}	133.044	0.564927	newly	10.	57.13	{3., 33.}	503.7	0.384261	Reff	0.729267	0.418523	{0.0922658, 0.62369}	57.53	ar	587.	1556.13	{1438., 1592.}	165.099
35	R0	{1.72889, 7.76068}	{2.78401, 5.09591}	{{2.45397, 0.143355}, {3., 15.5778}}	{61.2883, 60.7381}	inf	3.	58.1786	{6.44301, 90.5896}	1845.27	1.03636	newly	3.	94.08	{6., 208.}	3042.	1.06701	Reff	0.605112	0.591787	{0.183656, 0.793024}	54.415	ar	650.	1751.76	{1564., 2396.}	169.502
36	R0	{1.62644, 4.12303}	{2.56496, 7.47668}	{{1.38211, 0.911004}, {3., 14.2088}}	{66.7322, 101.687}	inf	0.1	50.6212	{7.82801, 55.4764}	50521.2	2.43294	newly	0.1	31.28	{6., 48.}	31180.	2.29835	Reff	0.566001	0.36701	{0.0525454, 0.566175}	50.3716	ar	652.	1673.59	{1559., 1931.}	156.686
37	R0	{1.70878, 15.8072}	{2.7406, 12.7945}	{{2.15996, 5.03588}, {3., 17.9903}}	{60.3832, 30.513}	inf	40.	44.6482	{10.2806, 100.267}	62.5933	0.315518	newly	11.	21.82	{4., 40.}	122.909	0.363696	Reff	0.659589	0.504481	{0.11719, 1.17121}	66.6533	ar	674.	1540.13	{1392., 1706.}	128.506
38	R0	{1.58967, 6.80568}	{2.69242, 7.43044}	{{2.0572, 0.521894}, {3., 15.8952}}	{71.9722, 67.8787}	inf	5.	47.3318	{6.0921, 49.8404}	852.483	0.726309	newly	3.	61.71	{4., 55.}	1963.67	0.871865	Reff	0.62474	0.527847	{0.151961, 1.20389}	65.4539	ar	610.	1723.73	{1550., 2110.}	182.579
39	R0	{1.344, 8.19498}	{2.64518, 7.46051}	{{1.73083, 1.69788}, {3., 19.4107}}	{96.8138, 78.268}	inf	14.	28.9723	{1.5102, 77.2842}	160.415	0.416104	newly	14.	52.63	{1., 91.}	340.214	0.552422	Reff	0.684096	0.565117	{0.0791194, 1.54455}	56.7431	ar	551.	1651.14	{1481., 2224.}	199.662
40	R0	{1.61509, 7.90825}	{2.4671, 7.38025}	{{1.25938, 4.47423}, {3., 12.1804}}	{68.0035, 31.8603}	inf	13.	67.2148	{9.74073, 61.0021}	429.441	0.532173	newly	9.	55.39	{6., 85.}	528.778	0.549668	Reff	0.7171	0.370569	{0.114398, 0.700098}	61.1227	ar	581.	1751.73	{1612., 1954.}	201.503
41	R0	{1.60017, 5.90605}	{2.57487, 4.09366}	{{1.42435, 0.489832}, {3., 11.8871}}	{66.1546, 72.675}	inf	6.	53.9022	{9.99854, 103.489}	801.642	0.767166	newly	6.	94.1	{11., 212.}	1470.33	0.86387	Reff	0.547258	0.547575	{0.209444, 1.03434}	54.2551	ar	661.	1735.65	{1566., 2008.}	162.579
42	R0	{1.64436, 15.1337}	{2.46311, 4.77539}	{{1.39245, 3.30417}, {3., 6.76563}}	{60.3348, 69.0169}	inf	40.	65.5582	{14.5206, 70.5109}	124.454	0.293051	newly	15.	94.97	{10., 118.}	546.333	0.437409	Reff	0.779427	0.544838	{0.136173, 1.2036}	61.4772	ar	640.	1751.64	{1554., 2104.}	173.694
43	R0	{1.48917, 11.917}	{2.33149, 6.17295}	{{0.731714, 3.65962}, {3., 9.34226}}	{85.4732, 48.7501}	inf	13.	43.0999	{5.65283, 28.0536}	252.859	0.369172	newly	9.	42.36	{4., 43.}	393.778	0.482624	Reff	0.775858	0.494285	{0.0769716, 0.816903}	53.4685	ar	551.	1523.93	{1367., 1997.}	176.575
44	R0	{1.65577, 7.89317}	{2.6593, 8.22445}	{{2.10364, 2.35876}, {3., 15.404}}	{63.4786, 46.5188}	inf	7.	68.2354	{14.463, 84.4764}	876.979	0.846784	newly	5.	60.	{11., 109.}	1104.	0.891247	Reff	0.738473	0.537993	{0.122361, 1.09064}	59.2301	ar	576.	1767.59	{1584., 2266.}	206.873
45	R0	{1.6909, 12.3275}	{2.39061, 5.49614}	{{0.28239, 1.49338}, {3., 11.0264}}	{76.6721, 62.128}	inf	23.	42.2009	{5.03348, 85.2624}	124.965	0.329887	newly	8.	34.18	{4., 83.}	347.75	0.546829	Reff	0.654378	0.357177	{0.045452, 0.650152}	69.3854	ar	675.	1673.52	{1529., 2019.}	147.929
46	R0	{1.62351, 16.0698}	{2.57909, 2.04884}	{{1.63405, 0.235938}, {3., 5.71089}}	{58.9038, 87.2504}	inf	51.	29.9083	{1.03346, 24.0937}	90.7829	0.711714	newly	13.	64.57	{1., 66.}	431.462	0.560342	Reff	0.899425	0.489942	{0.187382, 0.70365}	51.3319	ar	591.	1623.79	{1500., 1811.}	174.753
47	R0	{1.52372, 4.38599}	{2.39678, 4.71951}	{{1.12272, 1.92946}, {3., 7.45761}}	{73.3902, 36.1592}	inf	0.1	44.3574	{8.60541, 63.4629}	44257.4	2.36838	newly	0.1	54.05	{7., 89.}	53950.	2.36493	Reff	0.582061	0.415401	{0.100239, 0.825541}	57.1806	ar	618.	1722.1	{1566., 2017.}	178.657
48	R0	{1.44306, 11.788}	{2.77126, 3.64452}	{{2.21125, 0.967403}, {3., 13.5612}}	{92.0406, 74.1629}	inf	22.	32.3766	{3.39143, 46.9377}	105.214	0.348139	newly	12.	66.1	{5., 103.}	480.667	0.506628	Reff	0.722973	0.52372	{0.269264, 0.919437}	51.3616	ar	534.	1622.83	{1491., 1820.}	203.901
49	R0	{1.63624, 11.3893}	{2.66345, 7.65642}	{{2.10387, 1.8442}, {3., 17.8441}}	{66.5018, 56.2024}	inf	22.	58.5793	{11.5758, 83.7832}	186.951	0.358317	newly	9.	56.19	{9., 84.}	532.111	0.568289	Reff	0.623407	0.418613	{0.0258154, 0.851662}	56.8528	ar	643.	1665.59	{1537., 1890.}	159.034
50	R0	{1.46113, 11.1322}	{2.14303, 5.58372}	{{0.260532, 2.16716}, {3., 11.045}}	{96.6349, 49.8417}	inf	28.	30.1696	{1.95803, 47.2097}	109.24	0.584276	newly	17.	29.54	{2., 56.}	149.882	0.506458	Reff	0.831383	0.40429	{0.0459816, 0.603499}	68.8017	ar	501.	1615.4	{1504., 1853.}	222.435
